# 

**Published:** 1890-08-30

**Authors:** 


**Consumption: a Step Forward** - 321

**Words of Consolation.**—Wasted Opportunities - 322

**The Doctor's Pulpit.**—Cholera Prevention - 323

**Thoughts on Ill-Health.** III.—Moral Health - 324

**A Hospital in the Black Forest** - 325

**Turning Over New Leaves.**—Home Doctoring—The Deep Sea Fishermen - 326

**Everybody's Page** - 327

**Notes and News** - 328

**Annotations.**—Holidays and Health—A New Home for Epileptics—The Dangers of Tuberculous Milk - 330

**Passing Topics.**—Dangerous Lunatics at Large - 336

**The Practitioner's Mirror and Retrospect:—**

Medicine—Albuminuria and Life Assurance - 331

A Medical Museum—Foods, Drugs, Instruments, &c. - 332

Therapeutics—Use of Ether—Use of Aloes - 334

New Drugs, Appliances, and Things Medical—Knight's Antiseptic Eucalyptus Tablets - 334

The Practitioner's Bookshelf—Bayley's Pocket-Book for Pharmacists - 334

**Working Women in Large Towns.** VIII.—Shirt, Sack, and Umbrella Makers - 335

**Scraps and Gleanings** - 336

### EXTRA SUPPLEMENT—THE NURSING MIRROR.

**En Passant.**—Scarborough Institute—Work at Watford—Good News for Chertsey—Queen Victoria Nurses—A Day Off—American Directories—A Nurse in Trouble - xci

**Sketches of Insanity.** VIII.—Mental Stupor—Hypnotism - xcii

**Owed** (To a Charge Nurse) - xcii

**Why Hospital Authorities Object to the B.N.A.** - xciii

**The Patient** - xciii

**Notes and Queries—Presentation** - xciv

**Everybody's Opinion.**—Asylum Nurses—A Disclaimer—Life at Johannesburg, xciii; The Attack on the London Hospital—Registration of Midwives - xciv

**Vacancies** - xciv

**Amusements and Relaxation** - xciv

